# Invasive group A streptococcal disease surveillance in Canada, 2021–2022

**DOI:** 10.14745/ccdr.v50i05a03

**Published:** 2024-05-24

**Authors:** Alyssa R Golden, Averil Griffith, Gregory J Tyrrell, Julianne V Kus, Allison McGeer, Marc-Christian Domingo, Linda Hoang, Jessica Minion, Paul Van Caeseele, Hanan Smadi, David Haldane, Yang Yu, Xiaofeng Ding, Laura Steven, Jan McFadzen, Courtney Primeau, Kristyn Franklin, Irene Martin

**Affiliations:** 1 National Microbiology Laboratory, Public Health Agency of Canada, Winnipeg, MB; 2 Provincial Laboratory for Public Health, Edmonton, AB; 3 Public Health Ontario, Toronto, ON; 4 Department of Laboratory Medicine and Pathobiology, University of Toronto, Toronto, ON; 5 Toronto Invasive Bacterial Diseases Network (TIBDN), Department of Microbiology, Mount Sinai Hospital, Toronto, ON; 6 Laboratoire de santé publique du Québec, Institut national de santé publique du Québec, Sainte-Anne-de- Bellevue, QC; 7 British Columbia Centre for Disease Control, Vancouver, BC; 8 Roy Romanow Provincial Laboratory, Regina, SK; 9 Cadham Provincial Laboratory, Winnipeg, MB; 10 New Brunswick Department of Health, Fredericton, NB; 11 Queen Elizabeth II Health Science Centre, Halifax, NS; 12 Newfoundland and Labrador Public Health Laboratory, St. John’s, NL; 13 Queen Elizabeth Hospital, Charlottetown, PE; 14 Stanton Territorial Hospital Laboratory, Yellowknife, NT; 15 Yukon Communicable Disease Control, Whitehorse, YT; 16 Centre for Emerging and Respiratory Infections and Pandemic Preparedness, Public Health Agency of Canada, Ottawa, ON

**Keywords:** iGAS, *Streptococcus pyogenes*, Canada, *emm*, surveillance, antimicrobial resistance, group A *Streptococcus*

## Abstract

**Background:**

Invasive group A streptococcal (iGAS, *Streptococcus pyogenes*) disease has been a nationally notifiable disease in Canada since 2000. This report summarizes the demographics, *emm* types, and antimicrobial resistance of iGAS isolates collected in Canada in 2021 and 2022.

**Methods:**

The Public Health Agency of Canada’s National Microbiology Laboratory collaborates with provincial and territorial public health laboratories to conduct national surveillance of invasive *S. pyogenes*. *Emm* typing was performed using the Centers for Disease Control and Prevention *emm* sequencing protocol or extracted from whole-genome sequencing data. Antimicrobial susceptibilities were determined using Kirby-Bauer disk diffusion according to Clinical and Laboratory Standards Institute guidelines or predicted from whole-genome sequencing data based on the presence of resistance determinants.

**Results:**

Overall, the incidence of iGAS disease in Canada was 5.56 cases per 100,000 population in 2021, decreasing from the peak of 8.6 cases per 100,000 population in 2018. A total of 2,630 iGAS isolates were collected during 2022, representing an increase from 2021 (n=2,179). In particular, there was a large increase in isolates collected from October to December 2022. The most predominant *emm* type overall in 2021 and 2022 was *emm*49, at 21.5% (n=468) and 16.9% (n=444), respectively, representing a significant increase in prevalence since 2018 (*p*<0.0001). The former most prevalent type, *emm*1, increased from 0.5% (n=10) in 2021 to 4.8% (n=125) in 2022; similarly, *emm*12 increased from 1.0% (n=22) in 2021 to 5.8% (n=151) in 2022. These two types together accounted for almost 25% of isolates collected in late 2022 (October to December). Antimicrobial resistance rates in 2021 and 2022 included: 14.9%/14.1% erythromycin resistance, 4.8%/3.0% clindamycin resistance, and <1% chloramphenicol resistance.

**Conclusion:**

The increase of iGAS isolates collected in Canada is an important public health concern. Continued surveillance of iGAS is critical to monitor expanding *emm* types and antimicrobial resistance patterns.

## Introduction

Invasive group A *Streptococcus* (iGAS, *Streptococcus pyogenes*) is responsible for a wide range of human diseases, the most serious of which include bacteraemia, streptococcal toxic shock syndrome, necrotizing fasciitis, and endocarditis ((1)). In Canada, the overall incidence of iGAS infections has steadily increased since becoming a notifiable disease in 2000, peaking at a rate of 8.61 cases per 100,000 population in 2018 ((2)). In 2020, Canada reported decreased submissions of iGAS isolates, attributed to the containment measures put in place to control the SARS-CoV-2 pandemic (COVID-19) ((2)). There was also a significant shift in the *emm* types most commonly associated with disease in Canada, shifting from the formerly prevalent *emm*1 toward *emm*49 and *emm*76 ((2)).

In late 2022, the World Health Organization (WHO) reported that several countries in Europe had been observing increased cases of iGAS and scarlet fever, predominantly in children ((3)), starting off a season of increased focus on iGAS in many countries. As COVID-19 pandemic restrictions have loosened and person-to-person disease transmission has intensified, it is increasingly important to monitor the prevalence of both iGAS disease and associated *emm* types and antimicrobial resistance. This report provides a summary of iGAS isolates collected in Canada in 2021 and 2022.

## Methods

### Surveillance program

As previously described, surveillance of iGAS in Canada consists of a passive, laboratory-based system where invasive *S. pyogenes* isolates from all provincial and territorial public health laboratories (except Alberta) are forwarded to the National Microbiology Laboratory (NML) in Winnipeg for further testing ((2)). In 2021, a total of 2,179 iGAS isolates were reported, including 1,787 submitted directly to NML by provincial and territorial public health laboratories, as well as data for a further 392 isolates collected and tested by the Provincial Laboratory for Public Health in Edmonton, Alberta (ProvLab Alberta); in 2022, a total of 2,630 iGAS isolates were reported, including 2,108 submitted directly and data for 522 tested by ProvLab Alberta (**Table 1**). Sterile clinical isolation sites include blood, cerebrospinal fluid, deep tissue, biopsy and surgical samples, bone, and any clinical sources associated with necrotizing fasciitis or toxic shock syndrome.

**Table 1:** Number of invasive *Streptococcus pyogenes* isolates collected by each Canadian province/region, 2021–2022

Province	Age group (years)						Not given	Total
	<2	2–4	5–14	15–49	50–64	≥65		
**2021**								
British Columbia	2	1	2	153	125	76	0	359
Alberta	7	5	8	199	123	47	3	392
Saskatchewan	4	2	2	83	33	13	1	138
Manitoba	5	7	2	91	49	35	0	189
Ontario	9	1	8	352	227	176	7	780
Québec	7	5	3	90	73	57	2	237
Atlantic^a^	0	1	0	32	20	8	1	62
Northern^b^	2	1	1	4	11	3	0	22
**Canada**	**36**	**23**	**26**	**1,004**	**661**	**415**	**14**	**2,179**
**2022**								
British Columbia	6	4	7	151	147	109	1	425
Alberta	13	6	21	276	126	80	0	522
Saskatchewan	6	2	3	63	30	17	0	121
Manitoba	7	0	11	85	52	46	0	201
Ontario	8	13	23	315	258	282	6	905
Québec	15	12	27	134	87	90	0	365
Atlantic^a^	2	2	2	44	11	15	4	80
Northern^b^	0	0	0	7	2	2	0	11
**Canada**	**57**	**39**	**94**	**1,075**	**713**	**641**	**11**	**2,630**

^a^ Includes isolates from New Brunswick, Prince Edward Island, Nova Scotia, and Newfoundland and Labrador

^b^ Includes isolates from Yukon, Northwest Territories, and Nunavut

Population-based incidences of iGAS disease up to 2021 were obtained through the Canadian Notifiable Disease Surveillance System (CNDSS). Population data for incidence rates were obtained from Statistics Canada’s July 1^st^, 2021, annual population estimates.

### Isolate testing

*Streptococcus pyogenes* isolates were confirmed by a positive pyrrolidonyl-β-naphthylamide (PYR) reaction and susceptibility to bacitracin ((4)). From January 2021 to October 2022, *emm* typing was performed on all iGAS isolates submitted to NML and ProvLab Alberta using the Centers for Disease Control and Prevention (CDC)’s *emm* sequencing protocol available online. The sequences obtained were compared with the CDC *emm* database and results reported to the type level. Antimicrobial susceptibilities for iGAS during this time were determined using Kirby-Bauer disk diffusion for chloramphenicol (30 μg), erythromycin (15 μg), clindamycin (2 μg), penicillin (10 μg), and vancomycin (30 μg) according to Clinical and Laboratory Standards Institute (CLSI) guidelines ((5)). From November 2022 to December 2022, all iGAS isolates submitted to NML were whole-genome sequenced using the Illumina platform, with *emm* type identified directly using the WGS Analysis and Detection of Molecular Markers (WADE) pipeline. Antimicrobial resistance interpretation (susceptible, resistant) was also predicted using WADE, based on the presence/absence of resistance markers for: chloramphenicol (*cat*), macrolides/lincosamides (*ermA*, *ermB*, *ermT*, *mefA/E*) and β-lactams (*pbp2x*).

Supplementary testing was performed on all *emm*1 isolates submitted to NML in 2021–2022 to determine the prevalence of the novel M1_UK_ lineage. The M1_UK_ genotypes were determined by mapping whole-genome sequencing reads against reference strain MGAS5005 and identifying 27 characteristic genomic single nucleotide variants (SNVs), as previously described ((6,7)).

### Data analysis

Demographic data submitted with bacterial isolates included patient age, sex, clinical source, province, and date of collection. Multiple isolates with the same *emm* type and collected from the same patient within 14 days were counted once with the most invasive isolation site assigned. Meningitis-related isolates were regarded as most invasive, followed by blood, then other sterile sites. The laboratory data were aggregated by age into <2, 2–4, 5–14, 15–49, 50–64 and ≥65-year-old age groups, and regionally into Western (British Columbia, Alberta, Saskatchewan, Manitoba), Central (Ontario, Québec), Eastern (New Brunswick, Nova Scotia, Prince Edward Island, Newfoundland and Labrador), and Northern (Yukon, Northwest Territories, Nunavut) regions of Canada. Statistical significance of trends was assessed using the Cochran-Armitage test of trend, with a *p*-value of <0.05 considered to be statistically significant.

## Results

**Figure 1:**
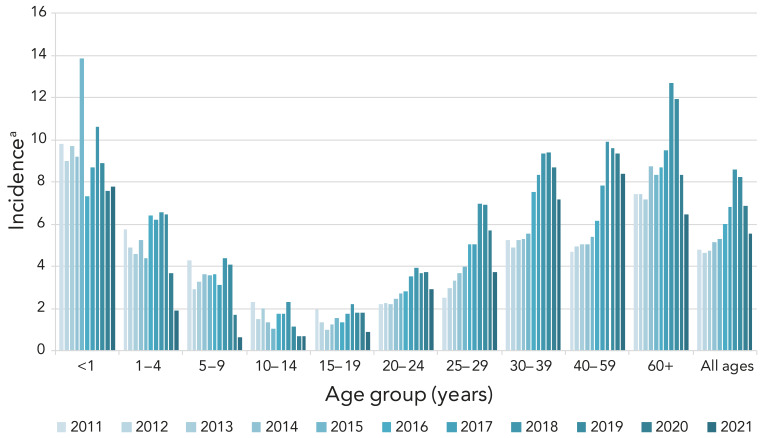
Annual incidence^a^ rates of invasive *Streptococcus pyogenes* cases in Canada, 2011–2021 ^a^ Cases per 100,000 population

**Figure 2:**
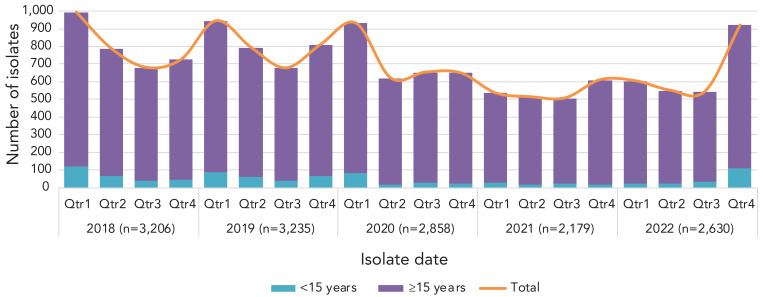
Number of invasive *Streptococcus pyogenes* isolates collected each quarter^a^ for children younger than 15 years and patients 15 years of age and older^b^, 2018–2022 Abbreviation: Qtr, quater ^a^ Qtr1, January to March; Qtr 2, April to June; Qtr 3, July to September; Qtr 4, October to December; all month ranges are inclusive ^b^ Yearly isolate counts include those where no age was given

After peaking at 8.61 cases per 100,000 population in 2018, the overall incidence of iGAS disease in Canada decreased in 2020 and 2021. The overall incidence rate in 2021 was 5.56 cases per 100,000 population, which is the lowest overall incidence in Canada since 2015 (**Figure 1**, **Appendix, Supplemental Table S1**). There was an increase in the number of iGAS isolates submitted in 2022 (n=2,630) in comparison to 2021 (n=2,179). In particular, there was a large increase in isolates collected in the final quarter (Q4; October to December) of 2022 (**Figure 2**), the total of which was considerably higher than Q4 in 2018 and 2019 (pre-pandemic years). Of note during 2022-Q4 it was an increased number of isolates collected from children younger than 15 years of age, in comparison to previous quarters.

The overall proportion of iGAS isolates collected from pediatric age groups remained stable over the two years, with infants <2 years of age accounting for 1%–2% of isolates, toddlers aged 2–4 years for 1%–1.5%, and children aged 5–14 years for 1%–3%. Proportions for other age groups had more fluctuation. Patients aged 15–49 years represented 46.1% of isolates collected in 2021 and 40.9% of those collected in 2022; adults aged 50–64 years 30.3% and 27.1%; and seniors aged 65 years and older for 19.0% and 24.4%. Of the isolates for which sex information was available, isolates from male patients represented 61.8% and 61.7% of isolates in 2021 and 2022, respectively. Blood was the predominant clinical isolation site, accounting for 69.3% of isolates collected in 2021 and 70.5% in 2022. Additional information on specimen source by age and *emm* type can be found in Appendix, **Figures S1–S5**.

**Figure 3:**
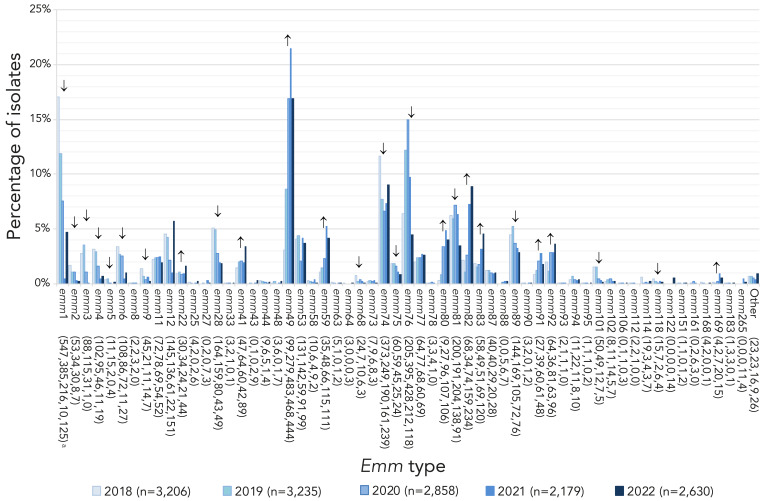
Prevalence of invasive *Streptococcus pyogenes emm* types in Canada, 2018–2022^a,b^ ^a^ Number of isolates for 2018, 2019, 2020, 2021 and 2022, respectively ^b^ For *emm* types with an overall (2018–2022) N≥30: up or down arrows indicate statistically significant trends toward increasing or decreasing prevalence for the 2018–2022 timespan, using the chi-squared test for trend. *Emm* types with no arrow either did not demonstrate a statistically significant trend, or did not have an overall N≥30

**Figure 4:**
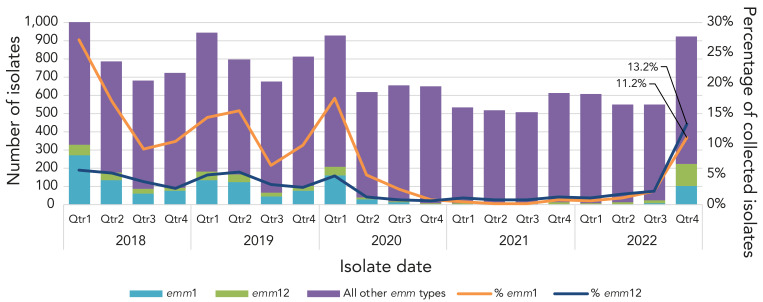
Number of invasive *Streptococcus pyogenes* isolates collected each quarter^a^ for *emm*1, *emm*12 and all other *emm* types^b^, 2018–2022 Abbreviation: Qtr, quater ^a^ Qtr1, January to March; Qtr 2, April to June; Qtr 3, July to September; Qtr 4, October to December; all month ranges are inclusive ^b^ Yearly isolate counts include those where no age was given

The most predominant *emm* type overall in 2021 and 2022 was *emm*49, at 21.5% (n=468) and 16.9% (n=444), respectively, representing a significant increase in prevalence since 2018 (from 3.1%, n=99; *p*<0.0001) (**Figure 3**). Other *emm* types that demonstrated significantly increasing trends from 2018 to 2022 include *emm*22 (0.9%–1.7%; *p*=0.025), *emm*41 (1.5%–3.4%; *p*<0.0001), *emm*59 (1.1%–4.2%; *p*<0.0001) *emm*80 (0.3%–4.0%; *p*<0.0001), *emm*82 (2.1%–8.9%; *p*<0.0001), *emm*83 (1.8%–4.6%; *p*<0.0001), *emm*91 (0.8%–1.8%; *p*<0.0001), and *emm*92 (2.0%–3.7%; *p*<0.0001). Other *emm* types demonstrated significantly decreasing trends (see Figure 3), such as *emm*1 from 17.1% (n=547) of all iGAS isolates collected in 2018 to 4.8% (n=125) in 2022 (*p*<0.0001). A percent prevalence of 4.8% in 2022 is a sharp increase from 2021, where *emm*1 only accounted for 0.5% (n=10) of isolates collected; this recent increase is statistically significant (*p*<0.0001). Of note, 49.0% (n=47) of *emm*1 isolates sequenced in 2022 were the novel M1_UK_ lineage; in comparison, in 2015 (the year the first M1_UK_ isolate was identified in Canada), only 2.6% (n=3) of sequenced *emm*1 isolates were M1_UK_. Another type of interest is *emm*12, which did not demonstrate a significant trend from 2018 to 2022; however, *emm*12 decreased significantly from 4.5% (n=145) in 2018 to 1.0% (n=22) in 2021 (*p*<0.0001), before significantly rising back up to 5.8% (n=151) in 2022 (*p*<0.0001). Counts of *emm*1 and *emm*12 saw a particular re-emergence in late 2022, together accounting for almost 25% of isolates collected in Q4 (**Figure 4**).

In 2021, the most common *emm* type from children <15 years of age was *emm*49 (28.2%, n=24). *Emm*49 dropped to the third most common type in this age group in 2022, instead replaced by *emm*12 (25.8%, n=49) and *emm*1 (24.2%, n=46) (Appendix, **Figure S6**). In patients aged 15 years and older, *emm*49 (21.3%, n=442) and *emm*76 (10.0%, n=207) were most common in 2021. In 2022, *emm*49 (17.0%, n=412) was also the most common type in the age group, followed by *emm*74 (9.7%, n=236) and *emm*82 (9.5%, n=230) (Appendix, **Figure S7**).

**Figure 5:**
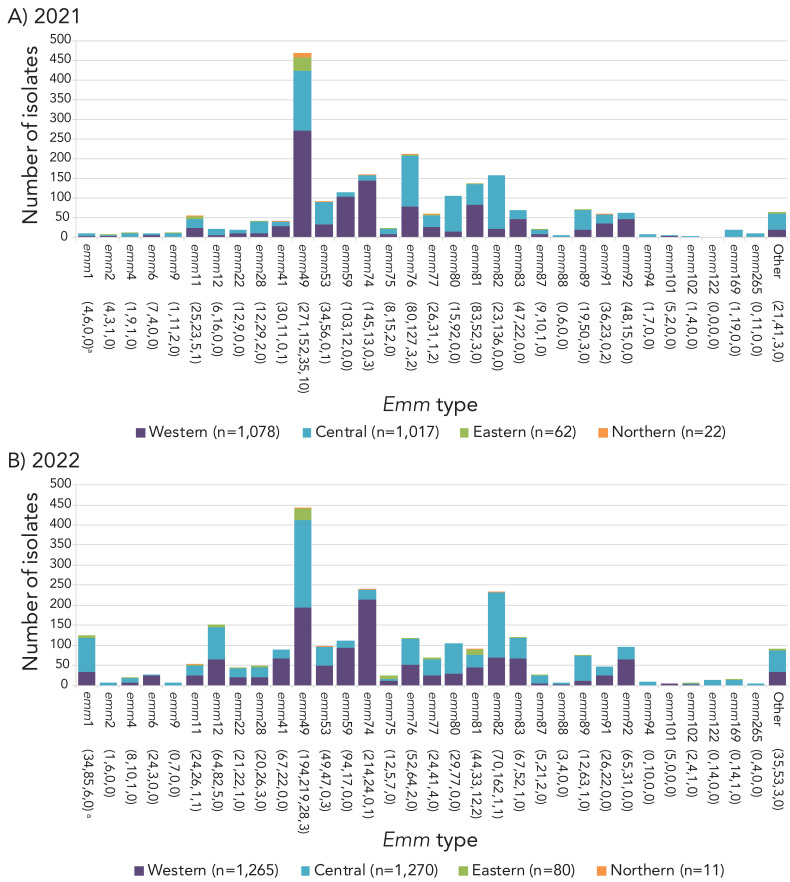
Regional distribution of invasive *Streptococcus pyogenes* isolates^a^ collected in A) 2021, and B) 2022, by *emm* type ^a^ Number of isolates in the Western, Central, Eastern, and Northern regions of Canada, respectively

*Emm* types associated with Western Canada (**Figure 5**) included *emm*49 (25.1%, n=271 in 2021; 15.3%, n=194 in 2022) and *emm*74 (13.5%, n=145 in 2021; 16.9%, n=214 in 2022). In Central Canada, *emm*49 (14.9%, n=152 in 2021; 17.2%, n=219 in 2022) and *emm*82 (13.4%, n=136 in 2021; 12.8%, n=162 in 2022) were predominant in both 2021 and 2022. *Emm*49 was the most common type isolated in Eastern Canada in both 2021 (56.5%, n=35) and 2022 (35.0%, n=28). Isolates from Northern Canada were highly represented by *emm*49 in 2021 at 45.5% (n=10), though only 22 isolates were submitted from this region. In 2022, only 11 isolates were submitted and there was no one common type (Appendix, **Figures S8–S11**).

Upon request, NML provides assistance to provincial and territorial public health laboratories for iGAS outbreak/case cluster investigations (including non-invasive isolates from screening) and jurisdictional *emm* increases. During 2021, NML assisted in four outbreak investigations from various jurisdictions, including *emm*53 (n=3 cases), *emm*76 (n=45), *emm*77 (n=2) and one multi-*emm* type outbreak (*emm*49 and *emm*53, n=8). An increased number of requests were received in 2022, where NML assisted with a jurisdictional increase (*emm*49) and seven outbreak investigations, including *emm*1.3 (n=3), *emm*41.11 (n=7 and n=9), *emm*49 (n=4), *emm*89 (n=23 and n=4) and two multi-*emm* type outbreaks (*emm*49, *emm*53, *emm*76, *emm*77, *emm*83.1, *emm*91 and *emm*169.3, n=20; *emm*6.4, *emm*41.11, *emm*49, *emm*59, *emm*74, *emm*75 and *emm*83.1, n=26).

**Figure 6:**
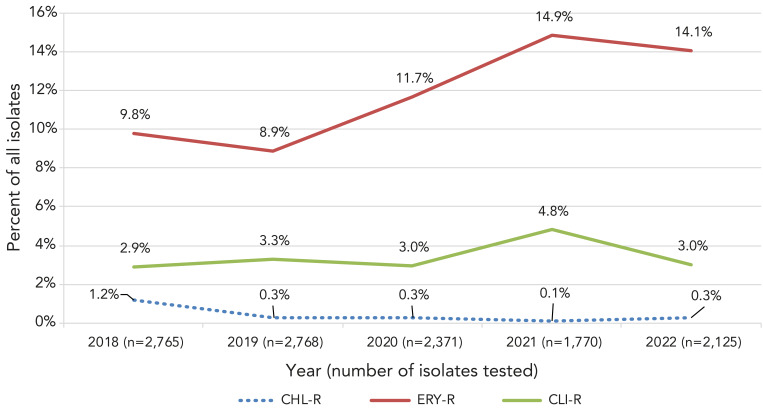
Antimicrobial resistance of invasive *Streptococcus pyogenes* in Canada, 2018–2022 Abbreviations: CHL-R, chloramphenicol-resistant; CLI-R, constitutively clindamycin-resistant; ERY-R, erythromycin-resistant

Antimicrobial resistance among iGAS isolates remained low in 2021–2022 (**Figure 6**, Appendix, **Table S2**). Erythromycin resistance increased significantly from 9.8% in 2018 to 14.1% in 2022 (*p*<0.0001), while chloramphenicol resistance decreased significantly from 1.2% to 0.3% (*p*<0.0001). Clindamycin resistance remained relatively stable over the study period (2.9%–4.8%). There was no resistance observed to penicillin or vancomycin. *Emm* types associated with erythromycin resistance and constitutive and inducible clindamycin resistance were similar in 2021 and 2022, including *emm*11 (88.9%/93.5% erythromycin resistance; 27.8%/22.6% constitutive clindamycin resistance; 66.7%/71.0% inducible clindamycin resistance); *emm*77 (92.3%/82.0%; 0%/0%; 92.3%/82.0%); *emm*83 (29.5%/42.7%; 4.9%/1.8%; 29.5%/42.7%) and *emm*92 (100%/95.5%; 0%/4.5%; 96.7%/69.3%) (Appendix, **Figures S12–S13**, **Tables S3–S4**).

## Discussion

In 2021, 2,127 cases of iGAS were reported to CNDSS, with a national incidence rate of 5.56 cases per 100,000 population, a considerably lower rate than the peak seen in 2018 (8.61 cases per 100,000 population). This low incidence in 2021 is consistent with the lower rate seen in 2020 (6.85 cases per 100,000 population) and can likely be attributed to indirect effects of the containment measures put in place in 2020 to prevent the spread of the SARS-CoV-2 pandemic virus (COVID-19). Numerous studies have observed that invasive bacterial disease activity due to pathogens transmitted by respiratory droplets (including *S. pyogenes*) decreased during this time ((2,8–10)).

Beginning in 2022, many countries began to see levels of iGAS disease increase once again. In December 2022, the WHO reported that five European countries had been observing increased cases of iGAS and scarlet fever, predominantly in children ((3)). Subsequently, the United States’ CDC advised of increased paediatric iGAS disease in several states, including Colorado, Minnesota, and Texas ((11–13)), and the Pan American Health Organization (PAHO) published an informative note urging member countries to remain watchful for iGAS cases after several were identified in Uruguay ((14)). In Canada, there was an increase in the number of iGAS isolates submitted to NML in 2022 in comparison to 2021. Though the total yearly count did not exceed the highest totals collected pre-pandemic (years 2018 and 2019), there was a large increase in isolates collected in 2022-Q4, including in children. The WHO indicated that the increase in iGAS infections may be due to increased population mixing following a period of reduced circulation of GAS during the COVID-19 pandemic, and increased circulation of respiratory viruses ((3)); respiratory viruses and viral co-infections are associated with GAS infections and may increase the risk of invasive disease ((3,15)). Though our current study is unable to provide any Canadian data on viral co-infections with iGAS, several studies, including those in France, the United Kingdom, and the United States, reported increased rates of viral infection prior to or concurrent with iGAS infections ((12,16,17)). Associated viruses included influenza, respiratory syncytial virus, SARS-CoV-2 pandemic virus, human metapneumovirus, and rhinovirus ((12,16,17)).

Of note, countries reporting an increase in paediatric iGAS disease in late 2022 universally identified *emm* types 1 and 12 as the predominant cause of cases ((12,13,18–21)). In Canada, prevalence of *emm*1 was decreasing considerably going into the COVID-19 pandemic and was virtually non-existent in 2021 (0.5% of collected isolates). Though *emm*1 counts remained relatively low at the beginning of 2022, the prevalence did increase in Canada in Q4, as was seen in other countries. Almost half of *emm*1 isolates tested in 2022 were the M1_UK_ lineage originally described by Lynskey *et al.*, as associated with hyperproduction of the SpeA exotoxin ((7)). Belgium, Netherlands, and the United Kingdom have also noted high rates (~75%) of the M1_UK_ lineage in 2022 ((22–24)). *Emm*12 has similarly been associated with toxigenic lineages; this type has previously been linked with outbreaks of scarlet fever, with associated lineages possessing exotoxin SpeC and superantigen SSA, as well as antimicrobial resistance ((25)). Prior to 2022, prevalence of *emm*12 was decreasing significantly in Canada. A large increase in prevalence in 2022-Q4 (just over 13% of all isolates collected), resulted in an increase to ~6% overall in 2022. Little antimicrobial resistance was seen in *emm*12 during that time. Studies in the United States (Colorado, Minnesota, Texas) also did not identify any resistance during their late 2022 increases of *emm*12 ((12,13)). In Portugal, the 2022 iGAS increase was characterized by *emm*12 isolates with high genomic diversity, with no expansion of a particular lineage ((20)). Further genomic characterization of *emm*12 isolates in Canada would be useful to identify toxin profiles and potential outbreak lineages.

The most common *emm* type collected in Canada since 2020 has been *emm*49. At the time of writing our previous annual report in 2020 ((2)), *emm*49 was not common in the literature as a frequent or emerging type. However, more recently, a study from the United States identified *emm*49 as increasingly associated with antimicrobial resistance. Li *et al*. have identified a macrolide and lincosamide-resistant sublineage of *emm*49 that has rapidly expanded in the state of Maryland to become the dominant lineage ((26)). A Spanish study also noted the emergence of *emm*49 in late 2022 after previously being rarely detected in the country. These isolates differed from the American lineage in that they demonstrated resistance to only tetracycline ((21)). Though antimicrobial resistance in *emm*49 was rarely detected in Canada in 2021 and 2022 (<2% erythromycin resistance), it will be important to monitor for the emergence of drug-resistant clones.

*Streptococcus pyogenes* remains susceptible to penicillin, the first-line antimicrobial treatment for iGAS infections, however, resistance to erythromycin (a second-line therapy) continues to increase in Canada. In 2021 and 2022, commonly collected *emm* types in Canada with high levels (>40%) of erythromycin resistance were similar to those reported in 2020, including *emm*11, *emm*77, *emm*83, and *emm*92 ((2)). Of these, *emm*83 and *emm*92 demonstrated significant increases over the 2018 to 2022 time period. Similar studies from other countries confirm that these *emm* types demonstrate resistance elsewhere, such as Spain (*emm*11, *emm*77) and the United States (*emm*11, *emm*83, *emm*92) ((26,27)). Of note is *emm*92, which was identified in West Virginia, United States, as an *emm* type with uniform resistance to macrolides/lincosamides that is disproportionately affecting patients with a history of intravenous drug use ((28)). In Canada, iGAS disease outbreaks often occur in at-risk groups, such as persons experiencing homelessness or those who abuse substances, closed populations such as long-term care facilities, and Indigenous communities ((29,30)); it will be of significant concern if drug-resistant *emm*92 continues to expand in Canada into vulnerable populations.

## Limitations

Caution should be exercised when interpreting the data presented in this report, as the overall interpretation of the results is limited to only isolates available for testing. Only a subset of the laboratory isolates from each province may have been submitted for testing, therefore, this report does not reflect the true incidence or rates of disease in Canada. The representativeness of the proportions of isolates submitted to NML for testing as compared to the CNDSS are presented in Appendix, **Table S5**. Not all provinces and territories report line list data to CNDSS, which means that only aggregated data are available at the national level. Therefore, CNDSS data and NML laboratory data are presented differently in terms of age grouping.

## Conclusion

Though the number of isolates collected was low in 2021, iGAS counts increased in 2022, particularly in the latter part of the year. *Emm*49 remained the most common type collected in Canada for 2021 and 2022; however, *emm*1 and *emm*12 began to rapidly increase in prevalence in the final quarter of 2022. As iGAS counts continue to rise following the COVID-19 pandemic, continued surveillance is imperative to monitor *emm* types and antimicrobial resistance in Canada. Enhancing surveillance to include linked epidemiological and laboratory data would improve our knowledge and interpretation of how iGAS *emm* types and antimicrobial resistance patterns affect at-risk groups in Canada.

## Appendix

Supplemental figures and tables are available upon request to the author.

Table S1: Annual incidence rates of invasive *Streptococcus pyogenes* in Canada by age group, 2011–2021

Figure S1: Clinical isolation sites of *Streptococcus pyogenes* from children younger than 15 years of age in A) 2021 (n=85) and B) 2022 (n=190)

Figure S2: Clinical isolation sites of *Streptococcus pyogenes* from patients 15 years of age or older in A) 2021 (n=2,094) and B) 2022 (n=2,436)

Figure S3: Percentage of invasive *Streptococcus pyogenes* isolates from blood in 2021 (n=1,509) and 2022 (n=1,853), by *emm* type

Figure S4: Percentage of invasive *Streptococcus pyogenes* isolates from other sterile sites in 2021 (n=664) and 2022 (n=769), by *emm* type

Figure S5: Percentage of invasive *Streptococcus pyogenes* isolates from cerebrospinal fluid in 2021 (n=6) and 2022 (n=8), by *emm* type

Figure S6: Prevalence of invasive *Streptococcus pyogenes emm* types isolated from patients less than 15 years old in 2021 (n=85) and 2022 (n=190)

Figure S7: Prevalence of invasive *Streptococcus pyogenes emm* types isolated from patients 15 years and older in 2021 (n=2,080) and 2022 (n=2,425)

Figure S8: Prevalence of the ten most common invasive *Streptococcus pyogenes emm* types collected from Western Canada in A) 2021 and B) 2022

Figure S9: Prevalence of the ten most common invasive *Streptococcus pyogenes emm* types collected from Central Canada in A) 2021 and B) 2022

Figure S10: Prevalence of the ten most common invasive *Streptococcus pyogenes emm* types collected from Eastern Canada in A) 2021 and B) 2022

Figure S11: Prevalence of the ten most common invasive *Streptococcus pyogenes emm* types collected from Northern Canada in A) 2021 and B) 2022

Table S2: Antimicrobial-resistant invasive *Streptococcus pyogenes* isolates by year, 2018–2022

Figure S12: Percentage of macrolide and lincosamide resistant *Streptococcus pyogenes* isolates collected in 2021, by *emm* type

Figure S13: Percentage of macrolide and lincosamide resistant *Streptococcus pyogenes* isolates collected in 2022, by *emm* type

Table S3: Percentage of macrolide and lincosamide resistant *Streptococcus pyogenes* isolates collected in 2021, by *emm* type

Table S4: Percentage of macrolide and lincosamide resistant *Streptococcus pyogenes* isolates collected in 2021, by *emm* type

Table S5: Number of invasive *Streptococcus pyogenes* isolates types by the National Microbiology Laboratory (NML) in comparison to the total number of cases reported to the Canadian Notifiable Diseases Surveillance System (CNDSS) in 2021, by patient age group
